# A practical guide to estimating treatment effects in patients with rheumatic diseases using real-world data

**DOI:** 10.1007/s00296-024-05597-2

**Published:** 2024-04-24

**Authors:** Are Hugo Pripp, Katarzyna Łosińska, Mariusz Korkosz, Glenn Haugeberg

**Affiliations:** 1 Oslo Centre of Biostatistics and Epidemiology, Oslo University Hospital, Oslo, Norway; 2 Faculty of Health Science, OsloMet – Oslo Metropolitan University, Oslo, Norway; 3 Division of Rheumatology and Immunology, University Hospital, Krakow, Poland; 4 Department of Rheumatology and Immunology, Jagiellonian University Medical College, Krakow, Poland; 5 Division of Rheumatology, Department of Internal Medicine, Sørlandet Hospital, Kristiansand, Norway; 6 Department of Neuromedicine and Movement Science, Faculty of Medicine and Health Sciences, NTNU, Norwegian University of Science and Technology, Trondheim, Norway

**Keywords:** Rheumatology, Regression analysis, Propensity score, Random Allocation, Observational studies as topic, Selection Bias

## Abstract

**Objective:**

Randomized controlled trials are considered the gold standard in study methodology. However, due to their study design and inclusion criteria, these studies may not capture the heterogeneity of real-world patient populations. In contrast, the lack of randomization and the presence of both measured and unmeasured confounding factors could bias the estimated treatment effect when using observational data. While causal inference methods allow for the estimation of treatment effects, their mathematical complexity may hinder their application in clinical research.

**Methods:**

We present a practical, nontechnical guide using a common statistical package (Stata) and a motivational simulated dataset that mirrors real-world observational data from patients with rheumatic diseases. We demonstrate regression analysis, regression adjustment, inverse-probability weighting, propensity score (PS) matching and two robust estimation methods.

**Results:**

Although the methods applied to control for confounding factors produced similar results, the commonly used one-to-one PS matching method could yield biased results if not thoroughly assessed.

**Conclusion:**

The guide we propose aims to facilitate the use of readily available methods in a common statistical package. It may contribute to robust and transparent epidemiological and statistical methods, thereby enhancing effectiveness research using observational data in rheumatology.

**Supplementary Information:**

The online version contains supplementary material available at 10.1007/s00296-024-05597-2.

## Introduction

Estimated treatment effects derived from Randomized Controlled Trials (RCTs) are considered the gold standard in study methodology and are required by regulatory authorities for approval and clinical use. RCTs aim to reduce bias in treatment effect results and enhance the accuracy of clinical investigations [1]. However, RCTs typically involve highly selected patients and adhere to strict inclusion and exclusion criteria, which may not accurately represent the heterogeneity of real-world patient populations. The risk of inadequate randomization, biased allocation, inconsistent analysis plans, and the influence of funding and other interests may bias findings and thus threaten the validity of RCTs [2]. Furthermore, causal conclusions of treatment effects in observational studies, particularly those using real-world data from clinical care, are less certain due to a lack of randomization and a number of both measured and unmeasured confounding factors. For instance, some patients may be more likely to receive one type of treatment (selection bias), thereby affecting the outcome. A naive statistical analysis of treatment effects using real-world data from patients with rheumatic diseases may therefore produce biased effect estimates.

Numerous studies in rheumatology assess treatment effects using a variety of study designs and analytical methods to minimize bias in observational studies based on real-world data. It is recommended that observational studies in rheumatology be designed and conducted in accordance with the recommendations outlined in the Strengthening the Reporting of Observational Studies in Epidemiology (STROBE) [3], and Good Research for Comparative Effectiveness (GRACE) guidelines [4]. The European Alliance of Associations for Rheumatology (EULAR) has endorsed overarching principles and points, including “robust and transparent epidemiological and statistical methods increase the trustworthiness of the results from observational data”, when analyzing and reporting comparative effectiveness research with observational data in rheumatology. EULAR also recommends that “authors should prepare a statistical analysis plan in advance” [5]. In our experience, the relationship between descriptive statistics, basic statistical tests, regression analysis, and the estimation of causal treatment effects can be somewhat ambiguous when these methods are used in research on rheumatic diseases. Therefore, a practical guide to estimating treatment effect in patients with rheumatic diseases using observational data could facilitate the aims outlined in both recommendations, guidelines, and principles. The need for such a guide is motivated from a study on effectiveness and persistence of biosimilar and reference etanercept in rheumatoid arthritis (RA) patients treated in ordinary clinical practice in Norway, where we compared the disease outcome using 28-joint Disease Activity (DAS28) and drug persistence between the unmatched patient cohort and the cohort matched using propensity score (PS) to adjust for confounders [6]. This approach was recently repeated in patients with psoriatic arthritis [7].

Ideally, the estimated treatment effect from observational data would uphold the required features of randomized trials, hence claiming comparable validity of RCTs and affecting clinical practice and patient care. Target trial emulation is a proposed methodological framework designed to achieve RCT-level validity of treatment effects on clinical outcomes when using observational data. This framework strives to develop a protocol for a hypothetical RCT and apply appropriate statistical methods. Thus, it addresses both the selection of eligible participants, treatment assignment, and statistical methods to adjust for confounding and selection bias common in observational data [8].

A recent review on statistical concerns in rheumatology research thoroughly outlines issues that arise in the analysis of disease cohorts and other sources. It emphasizes the need for careful analysis of observational data [9]. If the study design does not address causality, for example, through randomization, causal inference methods can be useful and may provide additional insights compared to classical statistical methods such as basic tests and regression analysis [10]. Causal inference quantifies the effect of treatment by comparing it under different conditions. The goal is to estimate causal effects based on a certain set of assumptions, with substantial applied, methodological and theoretical implications. Over the past few decades, significant progress has been made in statistical methods for causal inference, with the development of new estimators and tools such as Directed Acyclic Graphs (DAGs) and structural models for potential outcomes [11–13]. In addition to more methodological and mathematical work, recent tutorials have demonstrated the computational use of different causal inference estimators and how new estimators may address the limitations of the previous ones [14]. However, the theory and methods of causal inference for estimating treatment effects from real-world data might be somewhat challenging. The statistical notation and mathematical complexity of causal inference may impede the use of these methods in observational research and clinical care.

To address this issue, we present a nontechnical practical guide using a common statistical package (Stata) and a simulated dataset that mimics real-world data from patients with rheumatic diseases. This guide aims to facilitate the use of readily available methods to assess treatment effects in observational data, thereby aiding statistical analysis in clinical research in rheumatology.

## Causality and treatment effect

### Views on causality

In medical science and clinical practice, causality and treatment effects are both core concepts. Many viewpoints, principles and criteria have been developed to claim causal treatment effects. Among the most known are the Bradford-Hill criteria, proposed by the British epidemiologist Sir Austin Bradford Hill (1897–1991) in the early 1960s. This set of principles is used to assess the strength and causality of associations observed in epidemiological studies, considering factors such as strength of association, consistency, specificity, temporality, and biological gradient, among others [15]. It offers a systematic approach to draw causal conclusions from observational data.

Causal thinking has evolved since the Bradford-Hill criteria were established, building primarily on the potential outcome framework [16]. Rubin’s causal model and the counterfactual framework have formed a cornerstone in the field of causal inference, providing a systematic approach to quantifying causal effects in observational studies [17, 18]. To illustrate this way of thinking, consider a case where patients received either active treatment or a comparator (e.g., another drug, placebo or treatment as usual). If a particular patient received treatment (factual), then the same patient could not receive the comparator at the same time (counterfactual). This is the “fundamental problem of causal inference”. Unless we are able to travel back in time and reset everything that the patient had experienced before the treatment, it is impossible to compare the different outcomes of the same patient receiving these different treatments. In this framework, we nonetheless envision that a patient has two potential outcomes — the (observable) potential outcome of the factual treatment and the (unobservable) potential outcome of the counterfactual treatment (for example, the comparator condition). The causal effect of treatment on a patient is then defined as the difference between the potential outcome of the (factual) treatment and the potential outcome of the (counterfactual) comparator condition [19]. Rubin [17] extended this thought to the group level, defining the average causal effect as the difference between the average outcome of the people who received treatment and the average outcome of the people who received comparator treatment.

The Average Treatment Effect (ATE) and the Average Treatment Effect among the Treated (ATT) are two fundamental measures in the causal inference framework. ATE represents the average impact of the treatment across the entire population, measuring how the treatment influences outcomes on average. It addresses the question: What is the difference in the average outcomes between the whole population if they received the treatment and the whole population if they received the comparator condition? On the other hand, ATT specifically focuses on the subpopulation that receives the treatment. It measures the treatment effect for those who actually undergo it and addresses the following question: What is the expected difference in the potential outcome when the comparator condition is applied instead of the pivotal treatment condition [20]?

### Estimation strategies

To estimate the two potential outcomes, i.e., the potential outcome under the factual and the potential outcome under the counterfactual comparator treatment, one may specify the outcome, the treatment allocation, or a combination of those two. The main estimation strategies are regression adjustment (e.g., Stata command teffects ra), inverse-probability weighting (e.g., Stata command teffects ipw), and matching (e.g., Stata commands teffects nnmatch, teffects psmatch and the user-developed command psmatch2). To achieve statistical robustness, advanced estimation methods that combine regression adjustment and inverse-probability weighting (e.g., Stata commands teffects ipwra and teffects aipw) can also be used.

**Fig. 1:**
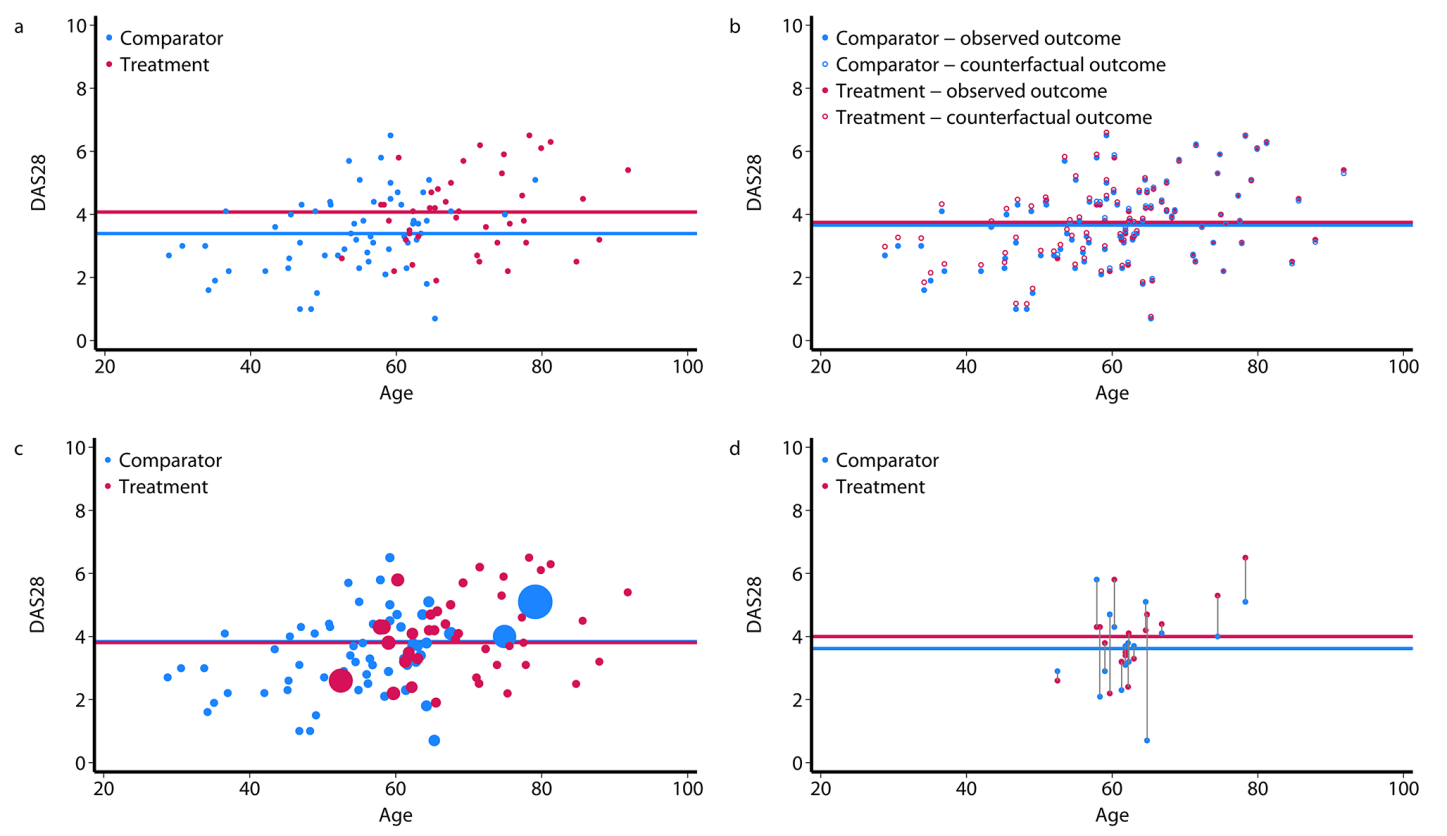
The estimation strategies are illustrated by **a**) raw data with unadjusted analysis, **b**) regression adjustment with observed and counterfactual outcomes, **c**) inverse-probability weighting with the size of markers proportional to weighting and **d**) one-to-one matching between comparator and treatment samples. The data are simulated

Figure 1 illustrates the abovementioned estimation strategies using a simulated dataset. The raw data and unadjusted statistical analysis showed an association between age and the Rheumatoid Arthritis 28-joint Disease Activity Score (DAS28) outcome and a substantial mean difference between the comparator and treatment groups (Fig. 1a). However, whether this substantial mean difference is due to a causal effect of treatment or simply confounded by the older age of the treatment group is questionable. Regression adjustment uses a regression model to estimate the potential outcome for each patient, i.e., the DAS28 for which a patient potentially received the other (counterfactual) treatment given a set of observed variables specified by a regression model. The hollow markers are the counterfactual potential outcome for each patient estimated from the regression model. Thus, we obtained a complete factual and counterfactual dataset to estimate the causal treatment effects (Fig. 1b). Another possible strategy is inverse-probability weighting, as shown in Fig. 1c. Rare observations within each treatment group (i.e., younger patients in the treatment group and older patients in the comparator group) get increased weights compared to more common observations (i.e., older patients in the treatment group and younger patients in the comparator group). The size of the markers represents the weight of each observation in the analysis. Finally, matching is a classical approach, and one-to-one matching has an intuitive appeal (Fig. 1d). Patients are matched on key characteristics (i.e., age in this simplified example). Thus, we obtained a dataset that was matched and balanced for confounding characteristics.

## Practical guide

### Data quality, variable selection and statistical model

Before analyzing data with statistical and causal inference methods, it is recommended that key elements of data quality be addressed. These include, among others as outlined by Hernán et al. [8] and Wang et al. [21]:

- Relevance: This includes relevant outcome measures, defined treatment groups, temporality, and important confounder variables.
- Data precision and quality: This involves the consistency of the data, the number of patients, the data capture mechanism and missing data.
- Data contemporaneity: This involves assessing whether the evolution of standards of care, clinical settings, and geographical representativeness makes the external data irrelevant.
- Other biases: It is important to eliminate other common sources of bias so that attention can be focused on confounders.
- Data flow: A CONSORT-like flowchart of eligible individuals to estimate the risk under each treatment strategy is recommended.

Medical and clinical expertise, along with available variables, often guides the selection of confounding covariates and the statistical model. A linear model is frequently the initial approach and is used in this practical guide, but one should consider nonlinear relationships and interactions between variables. Determining an absolute “correct” model is challenging, but we recommend assessing the effect of different models with increasing complexity, including machine learning methods [22]. This approach may ensure that the chosen model best fits the data and provides the most accurate and meaningful results.

### Dataset

To have a complete dataset without missing data, a known treatment effect and to avoid sharing sensitive patient data, we simulated a dataset that somewhat resembled the data structure in our previous work on real-world data. In contrast to our real-world data that motivated this guide [6], we assumed a stronger positive confounding effect of age and sex on treatment and DAS28 outcome. Furthermore, we assumed that there was no difference in the DAS28 outcome between the two treatment conditions after adjusting for confounding factors. The Stata codes for the simulated dataset are available as supplementary information, along with the complete dataset in Excel. In a study based on real-world data, it is recommended to investigate more confounding variables than DAS28 at baseline (variable name das28_0), age (variable name age) and sex (variable name sex) and to explore more complex statistical models than a simple linear effect. However, this can be easily integrated into our practical guide by changing the linear model das28_0 age i.sex with other variable combinations. This allows researchers to adapt the guide to their specific requirements and data.

### Univariable and multivariable regression analysis

Stata codes and selected outputs from univariable (curde) and multivariable (adjusted) regression analysis of treatment effects with the confounding variables DAS28 at baseline (das28_0), age and sex on DAS28 at follow-up (das28_1):

regress das28_1 i.treatment, base

margins i.treatment

margins i.treatment, pwcompare(effect)

**Table:** 

das28_1	Coefficient	Std. err.	t	*P* > t	[95% conf.	interval]
treatment						
0	0	(base)				
1	0.547	0.135	4.02	0.000	0.279	0.814
_cons	3.424	0.092	37.14	0.000	3.243	3.606

regress das28_1 i.treatment das28_0 age i.sex, base

margins i.treatment

margins i.treatment, pwcompare(effect)

**Table:** 

das28_1	Coefficient	Std. err.	t	*P* > t	[95% conf.	interval]
treatment						
0	0	(base)				
1	− 0.158	0.151	-1.05	0.295	− 0.457	0.139
das28_0	0.514	0.046	11.00	0.000	0.422	0.606
age	0.031	0.005	5.44	0.000	0.020	0.043
sex						
0	0	(base)				
1	0.089	0.118	0.76	0.450	− 0.143	0.323
_cons	− 0.402	0.374	-1.08	0.283	-1.139	0.333

A univariable linear regression of the two treatment groups yields identical results with an independent samples t-test, with an estimated coefficient of treatment (i.e., mean difference) of 0.54 (95% confidence interval (CI) 0.28 to 0.81). This is the “observed” treatment effect in the study, but in the presence of confounding variables, it is a biased estimate of the causal treatment effect. The coefficient of treatment from the multivariable model was − 0.15 (95% CI -0.46 to 0.14). In our example, the margin postestimation commands produce means with 95% CIs for each treatment group and mean differences between groups with 95% CIs from the statistical models. This postestimation is especially useful for complex statistical models, including interaction and nonlinear relationships.

### Regression adjustment

Stata codes for estimating the potential outcome means and average treatment effect by regression adjustment and linear regression adjusting for the confounding variable DAS28 at baseline, age and sex with selected outputs:

teffects ra (das28_1 c.das28_0 c.age i.sex, linear) (treatment), pomeans

teffects ra (das28_1 c.das28_0 c.age i.sex, linear) (treatment), ate

**Table:** 

das28_1	Coefficient	std. err.	z	*P* > z	[95% conf.	interval]
ATE						
treatment						
(1 vs. 0)	− 0.157	0.146	-1.08	0.282	− 0.443	0.129

regress das28_1 c.das28_0##i.treatment c.age##i.treatment i.sex##i.treatment

margins i.treatment

margins i.treatment, pwcompare(effect)

**Table:** 

	Contrast	std. err.	t	*P* > t	[95% conf.	interval]
treatment						
1 vs. 0	− 0.157	0.153	-1.03	0.306	− 0.458	0.144

The coefficients from the linear regression analysis (Stata command regress) are not directly interpreted as the mean difference or main effect between groups since the regression model contains several interaction terms. Thus, we show the postestimation using the margin command estimates of the marginal mean and mean difference between the groups from the statistical model. The estimates using regression adjustment (Stata command teffects ra) and regression analysis with selected interaction terms (Stata command regress) are simular, but with slightly different CIs. However, the interpretation of treatment effects after regression adjustment or regression analysis has a subtle difference. A regression model produces a conditional treatment effect (i.e., the average effect on the individual patient if covariates could be fixed), while causal regression adjustment produces an average treatment effect (i.e., the effect on the population using the potential outcome framework). For continuous outcome and linear regression, the difference is of more theoretical than practical importance due to identical effect estimates, but for binary outcome using logistic regression, effect results may differ due to the statistical property of noncollapsibility [23].

### Inverse-probability weighting

Stata codes and selected outputs for the potential-outcome means and average treatment effect by inverse-probability weighting:

teffects ipw (das28_1) (treatment das28_0 age i.sex, logit), pomeans

teffects ipw (das28_1) (treatment das28_0 age i.sex, logit), ate

**Table:** 

das28_1	Coefficient	std. err.	z	*P* > z	[95% conf.	interval]
ATE						
treatment						
(1 vs. 0)	0.132	0.158	0.83	0.406	− 0.179	0.443

logit treatment das28_0 age i.sex

predict p, pr

generate inv_p = 1/p if treatment = = 1

replace inv_p = 1/(1-p) if treatment = = 0

regress das28_1 i.treatment [pweight = inv_p], base

margins i.treatment

margins i.treatment, pwcompare(effect)

**Table:** 

das28_1	Coefficient	std. err.	t	*P* > t	[95% conf.	interval]
treatment						
0	0	(base)				
1	0.132	0.193	0.68	0.496	− 0.249	0.513
_cons	3.707	0.158	23.41	0.000	3.395	4.018

Estimating the treatment effect by inverse-probability weighting is achieved either directly by the causal function teffects ipw or indirectly by estimating the inverse probability of treatment using logistic regression followed by weighted linear regression. Both approaches produce identical effect measures but slightly different CIs. The probability score — or propensity — of exposure to the risk factor or intervention of interest is calculated, given an individual’s characteristics. Weights are then calculated as the inverse of the propensity score. The application of these weights to the study population creates a pseudopopulation with equally distributed confounders across exposed and unexposed groups [24]. In this pseudopopulation, we estimate the causal effect measures ATE and ATT.

### One-to-one propensity score matching

Stata codes for one-to-one propensity score matching with selected output for the corresponding paired t-test analysis:

psmatch2 treatment das28_0 age i.sex, outcome(das28_1) noreplacement odds logit neighbor(1)

label variable _das28_1 “DAS28 in matched comparator patients”

ttest das28_1 == _das28_1

**Table:** 

Variable	Obs	Mean	Std. err.	Std. dev.	[95% conf.	interval]
das28_1	138	3.971	0.101	1.188	3.771	4.171
_das28_1	138	3.491	0.102	1.208	3.287	3.694
diff	138	0.480	0.152	1.797	0.177	0.782

**Table:** 

mean(diff) = mean(das28_1 - _das28_1)		t = 3.1405
H0: mean(diff) = 0		Degrees of freedom = 137
Ha: mean(diff) < 0	Ha: mean(diff) != 0	Ha: mean(diff) > 0
Pr(T < t) = 0.9990	Pr(T > t) = 0.0021	Pr(T > t) = 0.0010

Several observational studies in rheumatology have used one-to-one propensity score matching [6, 25–28]. The user-developed Stata command psmatch2 implements this analysis with several options and variations. It can be installed in Stata with the command ssc install psmatch2. In our example, we omitted the restriction of differences in propensity scores for matching. However, it is recommended that researchers match within a limited propensity score difference. It not, samples with very different propensity scores and thus different confounding characteristics can be matched. One recommendation is matching limited to less than 0.2 of the standard deviation of the logit of the propensity score and then critically assessing the propensity score balance between the groups [29]. This affects the number of matching pairs and the estimation of treatment effects. In addition, the statistical propensity score model and matching with or without replacement may affect the results. One-to-one propensity score or other one-to-one matching methods intuitively resemble a RCT with an equal sample size and, ideally, similar patient (baseline) characteristics. However, it is disputed whether one-to-one matching indeed mimics RCTs. Since the propensity score method can only adjust for known and measured confounding factors, only RCTs may achieve an equal distribution of unknown confounders [30].

### Propensity score matching

Stata codes and with selected output for the average treatment effect by propensity score matching:

teffects psmatch (das28_1) (treatment das28_0 age i.sex, logit), ate

**Table:** 

das28_1	Coefficient	std. err.	z	*P* > z	[95% conf.	interval]
ATE						
treatment						
(1 vs. 0)	0.041	0.193	0.21	0.831	− 0.338	0.421

The Stata command teffects psmatch conducts somewhat more complete matching with reused observations and provides the possibility of more than one match to each treatment observation. The psmatch2 command can also perform this approach. While we find that the psmatch2 procedure is somewhat more “controllable”, the teffects psmatch command produced the expected ATE estimate of no difference and is in many ways easier to implement than the user-developed psmatch2 command.

### Double-robust methods

Stata codes and selected outputs for the potential outcome means and average treatment effect by the double robust estimators augmented inverse-probability weighting (AIPW) and inverse-probability-weighted regression adjustment (IPWRA):

teffects aipw (das28_1 das28_0 age i.sex) (treatment das28_0 age i.sex, logit), pomeans

teffects aipw (das28_1 das28_0 age i.sex) (treatment das28_0 age i.sex, logit), ate

**Table:** 

das28_1	Coefficient	std. err.	z	*P* > z	[95% conf.	interval]
ATE						
treatment						
(1 vs. 0)	− 0.072	0.152	-0.48	0.634	− 0.372	0.226

teffects ipwra (das28_1 das28_0 age i.sex) (treatment das28_0 age i.sex, logit), pomeans

teffects ipwra (das28_1 das28_0 age i.sex) (treatment das28_0 age i.sex, logit), ate

**Table:** 

das28_1	Coefficient	std. err.	z	*P* > z	[95% conf.	interval]
ATE						
treatment						
(1 vs. 0)	0.028	0.145	0.20	0.842	− 0.256	0.314

Double robust treatment effect estimators allow us to write a model for the treatment and a model for the outcome. Even if one of the models is misspecified, they are designed to produce robust estimates. Stata provides two double-robust estimators. AIPW implemented with the Stata command teffects aipw combines regression-adjustment and inverse-probability-weighted methods. IPWRA implemented with the Stata command teffects ipwra uses weighted regression coefficients to compute averages of treatment-level predicted outcomes, where the weights are the estimated inverse probabilities of treatment. Compared to those of previous examples, the mathematical and statistical properties of these methods are more complex. However, they offer a powerful tool for estimating treatment effects in observational studies, especially when there is a concern about model misspecification.

### Forest plot presentation

**Fig. 2:**
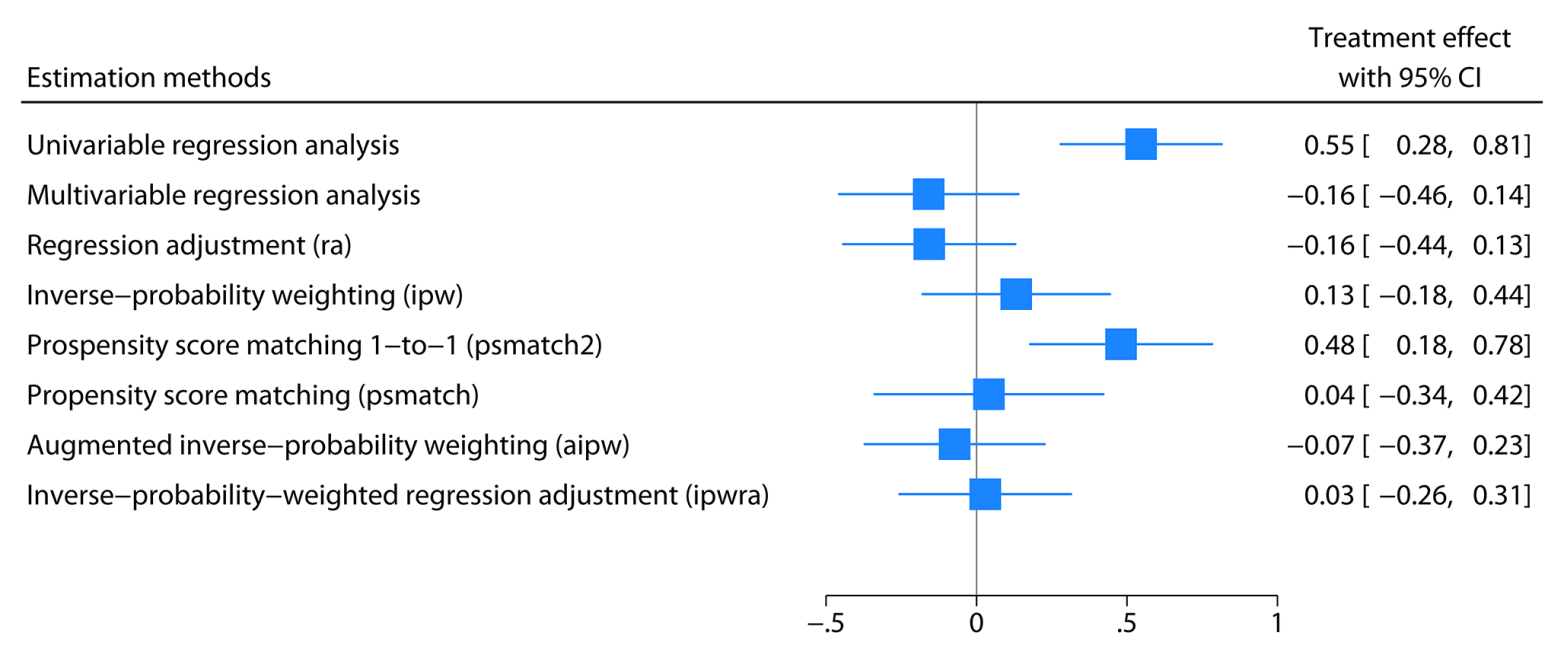
A forest plot made using the meta command in Stata summarizing the previous analyses

While working with real-world data, we can seldom affirm that one specific confounder variable model or estimator is correct. The number of confounders, their effects, and their statistical properties remain unknown; thus, analyzing the data with different models, variables, and estimation methods provides more insight and robustness. A forest plot is an efficient way to display results from several analyses and allows for easy comparison and interpretation of the results (Fig. 2).

**Fig. 3:**
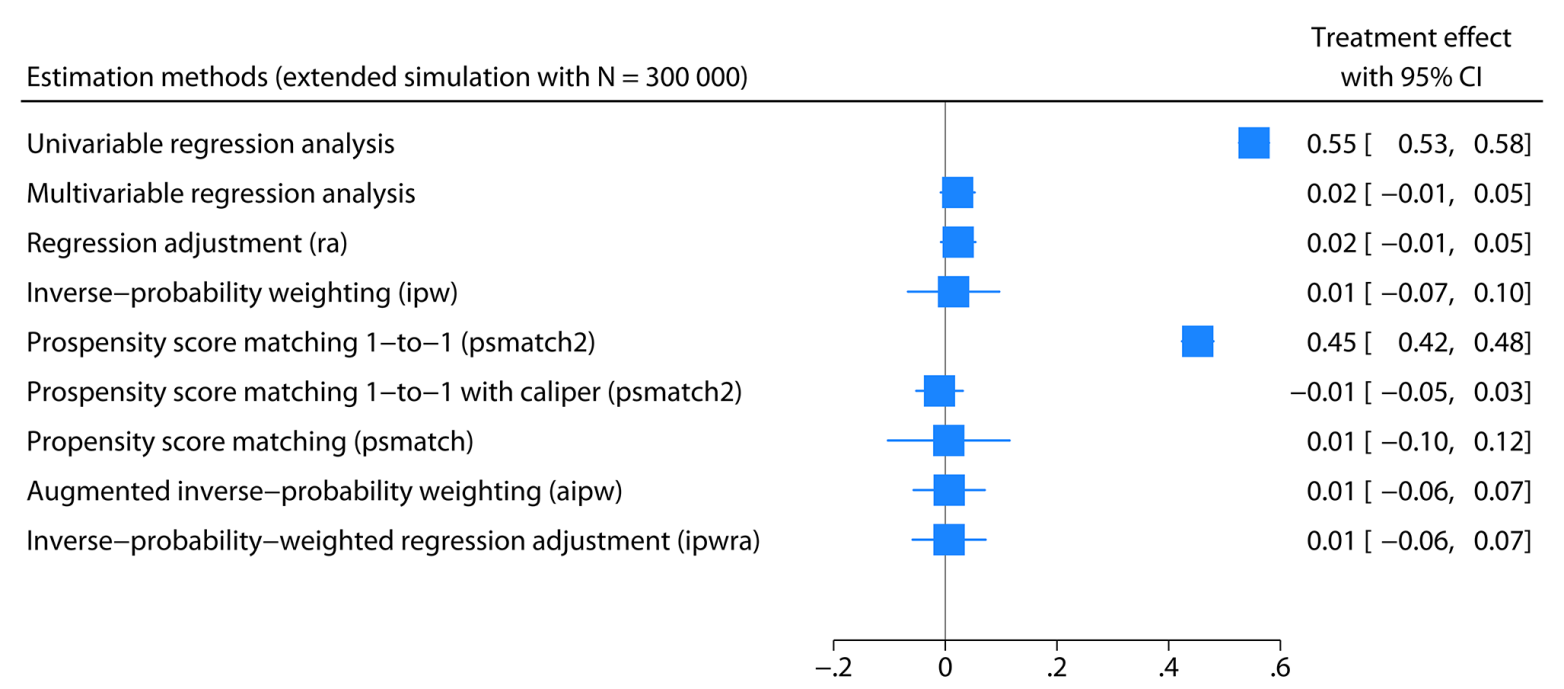
A forest plot summarizing an extended simulation. This simulation used a very high number of observations to limit random variation in the estimates

The simulated data assumed no causal relationship between treatment and DAS28 at follow-up, so we would expect no difference when adjusted for confounders. However, random noise and statistical variation can result in a numerical difference. All estimation methods produced different average treatment effects compared to the unadjusted analysis (univariable regression analysis). Simulating data with a very high number of observations is a simple approach to limit random variation in estimates [31]. Therefore, we replicated the same simulation procedure but with 300,000 observations (100,000 males and 200,000 females) and displayed the results in the forest plot in Fig. 3. All the evaluated estimation methods, except for one-to-one propensity score matching without restricting the possible difference in propensity score within each matched pair, produced the expected causal treatment effects (no difference between the groups). They “corrected” the naive approach using univariable regression analysis. One-to-one propensity score matching with recommended restrictions (with caliper), as outlined by Austin [32], produced the expected causal treatment effect.

## Recommendations.

Introducing causal inference methods to estimate treatment effects in rheumatic patients using real-world data adds a more through and robust analysis compared to using only common regression methods. The mathematical and statistical theory of causal inference offers complexity and challenges, but the integration of these methods and estimators in standard statistical software such as Stata could facilitate their use. This practical guide demonstrates several methods using a basic model with outcome at baseline, sex and age as confounders. By adapting the codes to other variables and extended models, the practical guide could serve as a template for hands-on analysis of treatment effects using real-world data. It may facilitate aims outlined in guidelines and recommendations for analyzing and reporting observational studies in rheumatology [3–5, 9].

A comparison of the estimator methods is beyond our scope, but our results indicate that the commonly used one-to-one propensity score matching methods should be supplemented with other estimator methods. In our example, one-to-one matching produced biased results compared to the expected findings in the simulated data. However, imposing restrictions on the matching procedure produced results similar to those of the other procedure. We further recommend outlining a statistical analysis plan for the selection of eligible patients and carefully assessing data quality on aspects such as missing data or sources of other biases. The choice of statistical methods should be guided by the research question, clinical knowledge, the nature of the data, and the assumptions of the methods.

### Electronic supplementary material

Below is the link to the electronic supplementary material.

Supplementary Material 1

Supplementary Material 2
