# Supplementary material for: A practical guide to estimating treatment effects in patients with rheumatic diseases using real-world data
Source: Rheumatol Int. 2024 Apr 24;44(7):1265–74. doi: 10.1007/s00296-024-05597-2 (PMC11178628; doi:10.1007/s00296-024-05597-2)
Supplement: Supplementary file 2 — Supplementary Material 2 [file 296_2024_5597_MOESM2_ESM.docx]

Supplementary information (SI)

Article title: A practical guide to estimating treatment effects in patients with rheumatic conditions diseases using real-world data

Journal: Rheumatology International

Are Hugo Pripp, PhD1,2 (ORICD 0000-0002-1678-7309); Katarzyna Łosińska, MD3,5 (ORICD 0000-0003-2801-6584); Mariusz Korkosz, MD, PhD3,4 (ORICD 0000-0002-1749-9739) and Glenn Haugeberg, MD, PhD5,6 (ORICD 0000-0002-9279-5191)

1. Oslo Centre of Biostatistics and Epidemiology, Oslo University Hospital, Oslo, Norway.
2. Faculty of Health Science, OsloMet – Oslo Metropolitan University, Oslo, Norway.
3. University Hospital, Division of Rheumatology and Immunology, Krakow, Poland.
4. Jagiellonian University Medical College, Department of Rheumatology and Immunology, Krakow, Poland.
5. Sørlandet Hospital, Division of Rheumatology, Department of Internal Medicine, Kristiansand, Norway.
6. NTNU, Norwegian University of Science and Technology, Department of Neuromedicine and Movement Science, Faculty of Medicine and Health Sciences, Trondheim, Norway.

Corresponding author: Are Hugo Pripp, e-mail: [apripp@ous-hf.no](mailto:apripp@ous-hf.no)

# Stata codes to simulate data

/* Age and DAS28 at baseline for males */

clear

matrix C = (1, .15 \ .15, 1)

corr2data das28_0 age, means(4 65) sds(1.2 13) corr(C) n(100) seed(1)

generate sex = 0

save "simulated data.dta", replace

/* Age and DAS28 at baseline for females */

clear

corr2data das28_0 age, means(4.2 60) sds(1.2 13) corr(C) n(200) seed(2)

generate sex = 1

replace das28_0 = 8 if das28_0 > 8

replace das28_0 = 1 if das28_0 <1

append using "simulated data.dta"

/* DAS28 at follow-up */

set seed 3

generate das28_1 = 0.5*das28_0 + 0.02*age + 0.05*sex + 0.3 +rnormal(0,1)

replace das28_1 = 10 if das28_1 > 10

replace das28_1 = 0 if das28_1 <0

/* Treatment allocation, label variables and patient id */

generate p = 1/(1+exp(-(0.25*das28_0 + 0.2*age + 0.4*sex - 14)))

generate treatment = rbinomial(1,p)

drop p

generate id = _n

order id, first

label variable id "Patient id"

replace das28_0 = round(das28_0, 0.1)

label variable das28_0 "DAS28 at baseline"

replace das28_1 = round(das28_1, 0.1)

label variable das28_1 "DAS28 at follow-up"

replace age = round(age, 0.1)

label variable age "Age"

label variable sex "Sex"

label variable treatment "Treatment"

save "simulated data.dta", replace
